# Assessment of the Contamination of Some Foodstuffs by *Escherichia coli* O157 in Benin, West Africa

**DOI:** 10.1155/2014/417848

**Published:** 2014-11-24

**Authors:** Honoré Sourou Bankole, Victorien Tamègnon Dougnon, Roch Christian Johnson, T. J. Dougnon, Boniface Yehouenou, Sylvain Kougblenou, Maxime Agonsa, Magloire Legonou, Thomas Dadie, Lamine Baba-Moussa

**Affiliations:** ^1^Department of Water and Food Hygiene, Ex-National Laboratory of Public Health, Ministry of Health, 01 P.O. Box, 418 Cotonou, Benin; ^2^Polytechnic School of Abomey-Calavi, Research Laboratory in Applied Biology, University of Abomey-Calavi, 01 P.O. Box, 2009 Cotonou, Benin; ^3^Interfaculty Center of Training and Research in Environment for the Sustainable Development, Laboratory of Hygiene, Sanitation, Toxicology and Environmental Health, Laboratory of Toxicology and Environmental Health, University of Abomey-Calavi, 01 P.O. Box, 1463 Cotonou, Benin; ^4^Polytechnic School of Abomey-Calavi, Research and Training Laboratory in Applied Chemistry, University of Abomey-Calavi, 01 P.O. Box, 2009 Cotonou, Benin; ^5^Training and Research Unit in Food Sciences and Technology, University of Nangui Abrogoua, 02 P.O. Box, Autoroute d'Abobo, 801 Abidjan, Cote D'Ivoire; ^6^Laboratory of Biology and Molecular Typing in Microbiology, Faculty of Sciences and Techniques, University of Abomey-Calavi, 05 P.O. Box, 1604 Cotonou, Benin

## Abstract

*Escherichia coli* O157 is a pathogenic bacterium causing haemorrhagic colitis. It represents a serious public health problem in Northern America and Europe, which can plague Africa. Most cases of mentioned poisoning were related to contaminated meat products and vegetables. The present work aimed to estimate the prevalence of *E. coli* O157 in meat and vegetables in Benin. For this purpose, 6 lots of faeces samples from pigs and 8 from cattle were collected at the farms on the outskirts of Cotonou. Similarly, 20 samples of carcasses, 20 samples of intestines and stomach, and 20 surfaces samples of slaughtering equipment were taken. Vegetables and environment materials in gardens have also been sampled for 84 samples. Bacteriological analyses revealed a percentage of contamination of 50% for pig faeces and 25% for cattle ones. All the meats from stalling parks have been contaminated by this bacterium. For vegetables, 14.6% of samples were contaminated by *E. coli* O157. The presence of this pathovar in animal breeding and slaughtering environment and in the gardens shows that Benin is not aware of the risks of foodborne illness associated with the consumption of contaminated products. Therefore, it urges including that germ in a systematic search during safety control of food products in Benin.

## 1. Introduction

Meat is considered as a privileged food because of its nutritive value. It is rich in proteins [1] and carries some essential amino acids. Meat is an important source of iron, vitamin B12, and lipids. Its nature makes it an indispensable food for a well-balanced food intake. Meat delivered for consumption comes from various animals like cattle, pigs, and goats [2]. Being convinced about the indispensable role that meat plays in food and nutritional security of the populations, Benin has granted a major place to the development of livestock keeping in its strategic plan of promotion of the agricultural sector implemented since 2008. However, in the future, consequences for Benin should be noted concerning food security in front of the emergence of new pathogens such as* E. coli,* which is a Shiga toxin producer.

In fact, since 1982, many cases of human infections following the consumption of food contaminated by the Shiga toxin of* E. coli* have been reported all over the world [3]. A record related to the association of meat products to the episodes of poisoning by* E. coli* O157 in the period of 1985 to 2001 showed, in the United States, 24 cases from 1985 to 1986, 73 cases from 1990 to 1992, 200 cases from 1997 to 1998, and 326 cases between 1999 and 2000. Canada, for its part, recorded 110 cases in 1990, United Kingdom 85 cases between 1994 and 1996, and France 105 cases from 2000 to 2001 [4].

Apart from the contamination related to the consumption of meat products, vegetables remained also problematic. They constitute excellent contributions of enrichment and diversification of human food. Vegetables are primary sources of minerals, vitamins, and other compounds that intervene in human nutritional health [5]. In Benin, market gardeners cultivate many vegetables. They require a high amount of nutrients. These nutrients can easily be used as inorganic fertilizers. However, there are numerous consequences related to their use. Among these, washing, erosion of soils, and pollution of the underground water can be mentioned [6]. This is the reason for which organic manures application is increasingly promoted. It provides nutritive elements to the plants and improves the soil structure [6, 7]. It has been reported that the application of organic manure, compared to the inorganic fertilizers, increases significantly the yield, for example, in the production of eggplants [6, 7].

Among these organic manures are the droppings of chicken, which are very prized in urban agriculture in Cotonou [8]. These faecal manures come back inexpensive to the market gardeners. In soil, they nourish the plant in major nutritive elements like nitrogen, phosphorus, and potassium. Nitrogen enhances the vegetation, accelerates the growth of the plant, and gives a good green coloration to the leaves. The phosphorus ions increase the development of roots and bulbs. They exercise an accelerating action on the maturity of the fruits, vegetables, and cereals. Potash makes the plant vigorous and allows it to resist the drought and diseases. The droppings are also a source of microelements such as magnesium, zinc, copper, sulphur, and boron. Apart from the nutritive elements, the decomposition of the droppings of chicken produces humus, which is highly useful for the consistency of soil that becomes soft and permeable to air and the roots [9]. Despite the advantages of the use of chicken droppings in urban agriculture, potential risks of contamination of the vegetables produced, related to the presence of pathogenic bacteria in the droppings, remain a preoccupation [10, 11]. The market gardeners, often hurried to produce and sell their products, do not master the sanitization of the chicken droppings. This highly water dependent activity is essentially assured by small-scale producers with poor income on reduced surfaces with rudimentary means. Nowadays, the land owning insecurity, the lower income of the producers, the poor water management, and the low level of organization of the stakeholders are some of the constraints that hinder the production of market cultures.

If it has been demonstrated in all those European countries that the recording of these food poisoning cases is related to poor food hygienic and handling practices, what would be the situation in Benin, where (1) no study was conducted on this pathovar up to date, (2) one relies on a quasi-non-existence of regulations in the meat sector, (3) the slaughterhouses do not meet the requirements, (4) people who are engaged in the activities of livestock keeping or meat products handling have no knowledge of good hygienic practices, (5) there is no regulation concerning the fertilization of soils with organic matters, (6) the cycle of maturation of the vegetables does not allow the market gardeners to respect one of the suggestions of the university of the state of Washington that stipulates that farmers must spread manure at least 60 days before the harvesting of all market products that will be consumed without prior cooking [12]?

Any answer to these questions could only be considered if supported by a scientific basis. This is why the present study was initiated and carried out on meat products and vegetables.

## 2. Materials and Methods 

### 2.1. Setting

The samples have been taken in the first time in the central slaughterhouse of Cotonou and on five raising farms numbered 1, 2, 3, 4, and 5. The first three farms are specialized in raising of cattle while the last two pigs. All five raising farms are situated in peripheral zone. In the second time, the withdrawals have also been achieved on the market site of Houéyiho, Cotonou. It is about the biggest market site at Benin. This site spreads on a surface of near 30 hectares and regroups more than 200 gardens. The choice of this site was motivated by the results of the works of Dougnon [11] that showed a high contamination of the droppings and vegetables by thermotolerant coliforms. The microbiological analyses were performed in the National Laboratory of the Ministry of Health (Benin).

### 2.2. Materials

#### 2.2.1. Samples Collected

The samples collected of the raising farms and the central slaughterhouse constituted of faeces, of intestines, of stomachs, and of swabbing of surface of the carcasses.

However, those collected at the market site of Houéyiho constituted of droppings of poultry, of the vegetables coming from the gardens using the droppings of poultry as fertilizer, and of the vegetables coming from the gardens using the NPK and the irrigation water. Tables 1 and 2 present an exhaustive point of the different samples used.

#### 2.2.2. Witness Source of* E. coli*

A witness source of* E. coli* O157 coming from an external assessment of the quality between the laboratory of the Ministry of Health (Benin) and a South African laboratory has been used in the present survey.

The used material is composed of laboratory facilities for microbiological use, reagents, and culture surroundings.

### 2.3. Methods

#### 2.3.1. Preparation of the Culture Surroundings, Soup, and Supplements

All culture surroundings, soup, and supplements used in the setting of this survey have been prepared and have been sterilized in accordance with the instructions of the manufacturers.

#### 2.3.2. Choice of the Gardens

The market gardens, where the droppings of poultry are used, were identified. 30 among them have been randomly kept at the rate of one by hectare and following a distance of at least 20 meters. On the 30 gardens, 3 have been selected by an uncertain pull. By garden, 4 samples of droppings, 4 samples of vegetables leaves (2 of* Solanum macrocarpon* and 2 of* Brassica oleracea*), 4 samples of carrots (*Daucus carota*), and 4 samples of irrigation waters have been taken. The same samples of the droppings of poultry have been collected outside in 3 gardens using the NPK solely as manure.

#### 2.3.3. Withdrawals

*Faeces*. The faeces have been collected in the central slaughterhouse and in 05 breeding farms, which contain 03 cattle breeding farms and 02 pigs breeding farms. With the help of a spatula, 250 g of faeces of the animals considered has been collected in a sterile sachet.

*Surfaces of Carcasses*. The withdrawal has been done by means of a sterile swab, moistened of soup modified tryptone soy, added from sterile novobiocin. The swabbing has been achieved on a surface of about 25 cm^2^ of the place aiming at the carcass of the animals considered. The used swab has finally been introduced in a tube containing 10 mL of soup modified tryptone soy added from sterile novobiocin.

For the carcass of cattle, the withdrawals have been done to the level of the necklace, the flank, the thigh, and the thorax, in the part close to the sternum with regard to external face, and to the level of the throat and the thigh for internal face.

For pig, the throat, the tether, the chest, the shoulder, and ham have been sampled as external face while the chest, ham, and the bib were sampled as internal face. 

*Intestines and Stomachs*. Using a pair of sterile scissors, 500 g of each of two organs of the animals considered has been appropriated in sterile sachets. 

*Surface of the Tables and Knives*. A surface of 100 cm^2^ of all tables and knives used before slaughtering of the considered animals has been appropriated by the technique of swabbing. The used swabs have been introduced each, in a tube containing 10 mL of soup modified tryptone soy added from sterile novobiocin. 

*Vegetables Leaves*. The vegetables leaves have been cut with the help of a pair of sterile scissors to 5 cm of the root. A mass of about 500 g cool leaves were thus appropriated on the plantations of vegetables and introduced in two sachets in plastic sterile. 

*Carrots (Daucus carota)*. The carrots have been uprooted to the hand after having worn sterile gloves. The leaves have been ridded with the help of a pair of sterile scissors and 500 g of carrots has been introduced in sachets in plastic sterile. 

*Droppings of Poultry*. With the help of a sterile spoon, 500 g of droppings of poultry was appropriated in sachets in plastic sterile. 

*Irrigation Waters*. The samples of irrigation waters were appropriated by immersion of the bottles in glass sterile of one liter in water.

All thus appropriated samples have been transported in an icebox to 4°C to the laboratory. The microbiological analyses have been done in the 4 hours that followed the withdrawals of the samples.

#### 2.3.4. Validation of the Method of Analysis

To validate the method of analysis, a strain of* E. coli* O157 from South Africa, obtained at the Medical Bacteriology Section of the National Laboratory of the Ministry of Health, was used. This strain was isolated from a faecal sample by the Section in the setting of a comparison of interlaboratory results. For this validation, the strain was first confirmed using the antiserum* E. coli* O157. Thereafter, a sample of grated carrots was artificially contaminated with* E. coli* O157,* Pseudomonas*,* Klebsiella*, and* E. coli*.

The analysis of this sample test was finally made while not only using the NF EN ISO 16654 standards but also experimenting several methods of culture. Cefixime-tellurite supplement was used at the stage of enrichment without using it for the selective isolation and then used only at stage of the selective isolation in the sorbitol MacConkey agar. Out of these two experiments, it was recorded that there were more colonies of* E. coli* O157 in the second case than in the first. Also, as described by Dadie [13], the culture of* E. coli* O157 was achieved at 37°C ± 1°C, 42°C ± 1°C, and 44°C ± 1°C on sorbitol MacConkey agar and CHROM agar O157. At the end of this preliminary experiment, a better growth of* E. coli* O157 at 37°C ± 1°C on sorbitol MacConkey agar and at 42°C ± 1°C on CHROM agar O157 was recorded. All these experimentations permitted observing the optimal conditions of culture of* E. coli* O157. The method of analysis below was then adopted.

#### 2.3.5. Treatment of the Samples

The treatment included a phase of preenrichment, a phase of enrichment, a phase of isolation, a phase of purification, a phase of biochemical identification, and a phase of serological identification.

**Figure 1:**
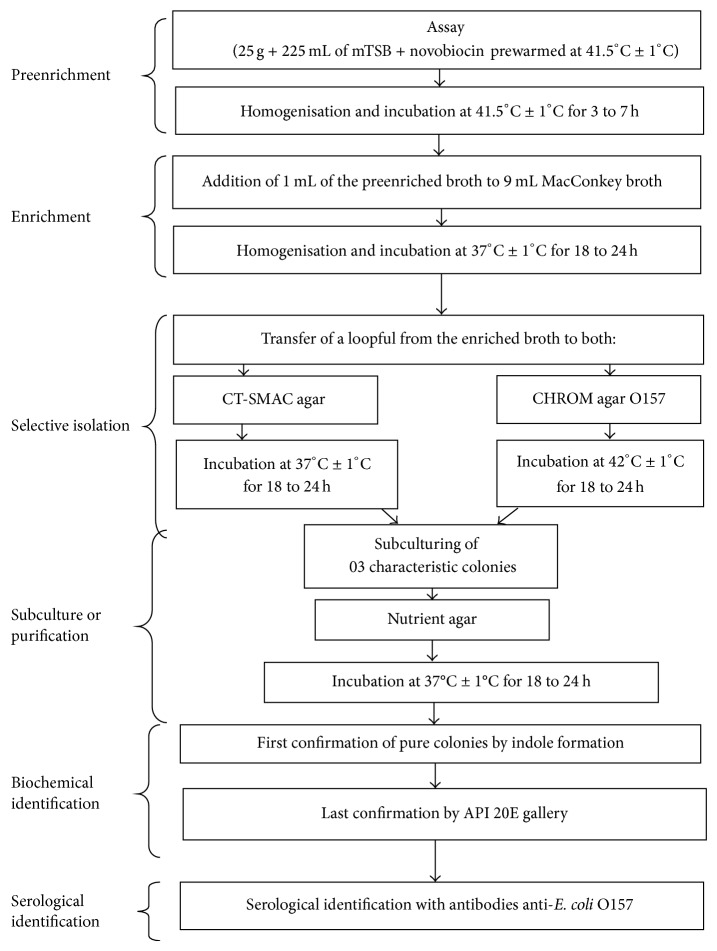
Method of treatment of the samples.

The method of analysis is summarized in Figure 1.

#### 2.3.6. Treatment of Waters

A volume of 100 mL of each of the water samples has been filtered to help in a membrane of porosity of 0,45 *μ*m.

#### 2.3.7. Treatment of the Stump Witness

The sample of grated carrots contaminated artificially with the stump reference acted as positive witness. This sample has been analysed in the same way to the other samples. The gotten result served to the validation of the results.

#### 2.3.8. Statistical Analysis

The analysed samples have been treated statistically with the help of Student *t*-test. While considering the different groups (cattle, pig, gardens using the droppings of poultry and gardens using nitrogen, and the phosphor and potassium), a doorstep of 5% (*P* < 0,05) has been defined for the values considered meaningful.

## 3. Results

On the 38 samples coming from the cattle, 02 were contaminated by* Escherichia coli* O157, with a rate of contamination of 5,26%.* E. coli* O157 has been put in evidence in 16,67% (1/6) of the faeces samples of cattle coming from the farms. As for the central slaughterhouse, the percentage of positivity is 50% (1/2). No sample not coming from the carcasses of cattle carried* E. coli* O157. In the same way the intestines, the stomachs of cattle, and the surfaces of tables and knives sampled were exempt of* Escherichia coli* O157 (Table 3).

*Escherichia coli* O157 has been recovered in 4 samples from pigs on the 36 analysed, with a rate of contamination of 11,11%, which contain 25% (1/4) of the samples of faeces coming from the farms and 100% (2/2) of the samples of faeces coming from the central slaughterhouse. The presence of* Escherichia coli* O157 has also been noted in 20% (1/5) of the samples of the external face of the pig carcasses. On the other hand* E. coli* O157 was absent in all samples of pig guts and in those coming from the tables and knives (Table 4).

The presence of* Escherichia coli* O157 has been revealed in 14,6% (7/48) of the samples appropriated in the first three gardens using the droppings of poultry as manure. It is necessary to note also that 8,3% (1/12) of the analysed droppings, 25% (3/12) of the vegetables leaves, 8,3% (1/12) of the samples of carrots, and 16,7% (2/12) samples of irrigation waters showed a positivity to the contamination of* Escherichia coli* O157 (Table 4).

While on the 36 different samples appropriated in the last three gardens using the NPK as fertilizer, no case of* Escherichia coli *O157 has not been detected (Tables 5 and 6).

## 4. Discussion

Previously remarked in the qualitative appreciation of meats and meat products, the emergence and the importance of the* E. coli* O157 are in clear progression regarding the cases of infections and in correlative manner with the deaths that occurred to the United States and Europe since the years 1985 to 2001. Studies performed in Australia by Cockerill III et al. [14] and Cobbold and Desmarchelier [15], in Cameroon by Cunin et al. [16], in Nigeria by Okeke et al. [17], and in Democratic Republic of Congo by Koyange et al. [18] showed that this microorganism was initially detected in livestock environment and in meats and meat products. The results of the present study confirmed those of the aforementioned authors.

In fact, in the first time, on the 38 samples coming from the cattle, 5,26% proved to be positive to this microorganism. As for the samples coming from pigs, 11,11% proved to be positive on the 36 samples used.

The microbiological works of investigating of detection and identification of this bacterium were notorious in the western countries and have drawn very early on the alarm as for the unusually virulent character of this microorganism. Indeed according to the literature [13], it is responsible for a hemolytic and uremic syndrome, of thrombocytopenia and of renal insufficiency in man.

In the results of the present survey, it comes out again globally on the one hand that* Escherichia coli* O157 has been recovered in the samples of faeces of the two animal species with respective percentages of 16,67% and 50% in the cattles in the farms and in the central slaughterhouse and 25% and 100% in pigs according to the same localizations. On the other hand, the impact of this microorganism was not the same in the samples of meats coming from each of the two animal species. Indeed, if the microorganism was completely absent in the samples of meat of cattle, it was not the same thing for the samples coming from the pig meat (10% on the carcasses). The total absence of this microorganism on the carcasses of cattle does not exclude the risk of to recover it in meat.

Besides, its presence in the faeces poses entirely the problem of the gentleness of the manipulations and the observance of the rules fundamental of the insurance quality during the operations of slaughtering and all unit operations some passing by the evisceration and the cleaning of meat before its stake some consumption. Indeed the works of Nou et al. [19], of Barkocy-Gallagher et al. [20], of Bosilevac et al. [21], and of Arthur et al. [22] on the one hand in the United States and on the other hand those of Fegan et al. [23] in Australia showed the presence of* Escherichia coli* O157 on the carcasses of cattle. There is the potential risk therefore to meet some if the leading measures of hygiene and healthiness during the manipulation are not respected.

The presence of* Escherichia coli* O157 in the samples coming from the carcasses of pigs could sometimes be bound to the behavior omnivorous of this animal species that sometimes makes use of its consumption of the faeces of all nature, which could confer possible contamination of meat by the microorganisms descending from them which have* Escherichia coli* O157.

If one considers manner of the results descending from all samples appropriated on every group of animals, while putting those of faeces, of the carcasses, and of the materials, the probabilities of detection, of* Escherichia coli* O157, are, respectively, 5,26% and 11,11%, for the samples coming from the cattle and pigs, very meaningful percentages to the doorstep of acceptability of 5% (*P* < 0,05), which implies that the risk of the presence of* Escherichia coli* O157 exists at the two animal species and manner more raised in the samples coming from pigs. This fact justifies therefore that the environment of raising constitutes a source of contamination of meats, of cattle, and of pigs by* Escherichia coli* O157 in Benin.

In the second time, on the 48 samples, coming from the gardens amended by the droppings of poultry, 14,6% were revealed positive to this microorganism. On the other hand, none of the samples coming from the gardens not using nitrogen-phosphor-potassium showed the presence of this bacterium even though it is likely that this bacterium is localized in the soils of culture. This first observation poses the problem of hygienisation of the droppings before their epandage in the market gardens. Indeed, none of the market gardeners of the gardens in which the present work took place do not proceed to the hygienisation of the droppings before use. Indeed, this process permits one considerable reduction of the load of the droppings in microorganisms, especially of the coliforms which have* Escherichia coli* O157.

Besides, the analyses done on the irrigation waters showed that they also transport this microorganism to 8,3%, which explains therefore that the vegetables coming from the gardens using nitrogen-phosphor-potassium are not safe as much as from a contamination by* Escherichia coli* O157; it is sufficient that these vegetables are irrigated by a water contaminated by this microorganism. It is necessary to note that the point of irrigation water was adjoining to an enclosure of raising of pigs, which could be the basis of the contamination of this water. Indeed, several works showed that the ovine constitutes outside of the bovine, a source of contamination in* Escherichia coli* O157. It is the case of the results of the works of Keen et al. [24], achieved in 2006 on 1102 samples of faeces of pigs analyzed during a fair of livestock regrouping 32 states of the United States, who showed that 1,2% of the samples were contaminated by* Escherichia coli* O157: H7, 0,8% by* Escherichia coli* O157: H7, nonproducers of Shiga toxins, and 1,7% by* Escherichia coli* non-O157 H7, nonproducers of Shiga toxins.

In total, on the 1102 samples of pork stools analyzed, 41 were contaminated by* Escherichia coli* O157, with a percentage of 3,7%. These works keenly confirm the logic therefore according to which the vegetables leaves would have undergone a contamination crossed by the irrigation water, certainly contaminated by the faeces of pigs. In the same way, this contamination of the vegetables leaves could be due to the droppings of poultry used, insofar as the rate contamination of them has been valued to 8,3%.

In garden 3 contrary to garden 1, the vegetables leaves of* Solanum macrocarpon* were not contaminated by* Escherichia coli* O157, which is not the case of the leaves of* Brassica oleracea*. This report would be bound on the one hand to the architecture of the vegetables of* Solanum macrocarpon* and on the other hand to the particular disposition of the* Brassica oleracea* leaves. That disposition confers them a capacity of retention of water contrary* Solanum macrocarpon*. This retention of water encourage the survival of* Escherichia coli* O157 during several days. These hypotheses are supported by the works of Abdul-Raouf et al. [25] who have demonstrated in 2005 the capacity of* Escherichia coli* O157 to develop itself between 12 and 21°C on the one hand and those of Rice et al. [26] who demonstrated in 1992 the survival of 2 stumps of* Escherichia coli* O157 to 20°C during 40 days on the other hand. Also, irrigation waters of marsh were of watering used in the gardens. The practice of raising in gardens 1 and 3 contributed to this situation of the presence of* Escherichia coli* O157 in samples of these gardens, because waters of surface and the waters of marsh are in this case some potential sources of contamination in* Escherichia coli* O157. That situation confirmed the work of Heijnen and Medema [27] that revealed the presence of* Escherichia coli* O157, in all the samples from surface and worn-out waters.

The presence of* Escherichia coli* O157 in the samples of droppings and of carrots of garden 2 and its absence in the samples of water contrary to those of* E. coli* O157, in all samples of waters of surface and of analyzed worn-out waters of gardens 1 and 3, show that the samples of carrots would have been contaminated by the contaminated droppings. It is the water that would have acted here as vector of* Escherichia coli* O157, of the droppings toward the carrots. Indeed, in garden 2, the withdrawals took place 15 minutes after the watering whereas water had been revealed exempt of* Escherichia coli* O157.

Globally if one takes into account the two potential sources of contamination, droppings and irrigation waters, the middle value of the risk of contamination of* Escherichia coli* O157 is 8,3%, very meaningful percentage by report to the doorstep of acceptability of 5% (*P* < 0,05), which implies that the risk of the presence of* Escherichia coli* O157 exists in the market gardens implied in this survey. The obtention of strains of* Escherichia coli*, others than those of* Escherichia coli* O157, would certainly be bound to the method of research used. It therefore showed the weakness of the performance of this medium. In fact, it shows the importance for associate a second more effective medium, other than CHROM Agar O157. This survey comes to complete the works of Onoue et al. [28] therefore achieved in 1999 that recommend the use of the culture surroundings containing a substratum of the *β*-glucuronidase for the isolation of* Escherichia coli* O157. This survey comes to complete those of other authors also as Dadie in 2001 that have shown that the classic process of culture and setting in evidence of* Escherichia coli* that recommends an incubation to 44-45°C does not encourage a development optimal of* Escherichia coli* O157. Indeed, during the validation of the method of analysis,* Escherichia coli* O157 knew a more abundant growth to 42°C and to 44°C on the CHROM agar gelose as Dadie raised it. Also, Doyle and Schoeni [29] in 1994 expressed that* Escherichia coli* O157 does not resist the usual conditions of treatment of food, which would explain their weak development to 44,5°C.

The detection of* Escherichia coli* O157 in the different types of analyzed samples shows that it is henceforth possible to search for, even in routine, this microorganism in food in Benin.

## 5. Conclusion

This survey shows that the level of hygiene is still poor in the market gardens, especially considering the cultural practices. It also demonstrates the risks related to meat products in Benin. Isolated for the first time in Benin,* E. coli *O157, which is emphasised in the food safety control in several western countries, exists as well in our country. Therefore, there is a potential risk of food poisoning by* E. coli *O157 to the farmers, the market gardeners, and Benin consumers.

## Figures and Tables

**Table 1:** Situation of samples collected.

Samples	Cattle	Pigs	Number	
	*n*	*n*	*n*	(%)
Faeces	08	06	14	(18,91)
Surfaces of carcasses	10	10	20	(27,78)
Intestines	05	05	10	(13,89)
Stomachs	05	05	10	(13,89)
Surfaces of tables	05	05	10	(13,89)
Surfaces of knives	05	05	10	(13,89)

Total	38	36	74	(100,00)

**Table 2:** Situation of the samples collected on the market site.

Samples	Number	%
Dropping of poultry	12	14,29
Vegetables leaves	24	28,57
Carrots (*Daucus carota*)	24	28,57
Irrigation water	24	28,57

Total	74	100,00

**Table 3:** Results of analyses of the samples descending from the cattle.

Samples	Results		Total
	Positive	Negative	
Faeces			
Raising farms	**01 (16,67%)**	05 (83,33%)	06 (100%)
Slaughterhouse	**01 (50%)**	01 (50%)	02 (100%)
Surfaces of carcasses	**00** (00%)	10 (100%)	10 (100%)
Intestines	00 (00%)	05 (100%)	05 (100%)
Stomachs	00 (00%)	05 (100%)	05 (100%)
Surfaces of tables	00 (00%)	05 (100%)	05 (100%)
Surfaces of knives	00 (00%)	05 (100%)	05 (100%)

Total	**02 (5,26%)**	36 (94,74%)	38 (100%)

**Table 4:** Results of analyses of samples descending from the pig.

Samples	Results		Total
	Positive	Negative	
Faeces			
Raising farms	**01 (25%)**	03 (75%)	04 (100%)
Slaughterhouse	**02 (100%)**	00 (00%)	02 (100%)
Surfaces of carcasses			
External face	**01 (20%)**	04 (80%)	05 (100%)
Internal face	00 (00%)	05 (100%)	05 (100%)
Intestines	00 (00%)	05 (100%)	05 (100%)
Stomachs	00 (00%)	05 (100%)	05 (100 %)
Surfaces of tables	00 (00%)	05 (100%)	05 (100%)
Surfaces of knives	00 (00%)	05 (100%)	05 (100%)

Total	**04 (11,11%)**	32 (88,89%)	36 (100%)

**Table 5:** Summary of the results of the samples of gardens 1, 2, and 3 using droppings of poultry.

Samples	Results		Total
	Positive	Negative	
Droppings poultry	**01 (8,3%)**	11 (91,7%)	12 (100%)
Vegetables leaves	**03 (25%)**	09 (75%)	12 (100%)
Carrots	**01 (8,3%)**	11 (91,7%)	12 (100%)
Irrigation waters	**02 (16,7%)**	10 (83,3%)	12 (100%)

Total	**07 (14,6%)**	41 (85,4%)	48 (100%)

**Table 6:** Summary of the results of the samples of gardens 4, 5, and 6 using NPK as fertilizer.

Samples	Results		Total
	Positive	Negative	
Vegetables leaves	00 (00%)	12 (100%)	12 (100%)
Carrots	00 (00%)	12 (100%)	12 (100%)
Irrigation waters	00 (00%)	12 (100%)	12 (100%)

Total	00 (00%)	36 (100%)	36 (100%)
